# Killer Archaea: Virus-Mediated Antagonism to CRISPR-Immune Populations Results in Emergent Virus-Host Mutualism

**DOI:** 10.1128/mBio.00404-20

**Published:** 2020-04-28

**Authors:** Samantha J. DeWerff, Maria A. Bautista, Matthew Pauly, Changyi Zhang, Rachel J. Whitaker

**Affiliations:** a Department of Microbiology, University of Illinois, Urbana-Champaign, Urbana, Illinois, USA; b Infection Genomics for One Health Theme, Carl R. Woese Institute for Genomic Biology, University of Illinois, Urbana-Champaign, Urbana, Illinois, USA; University of California, Irvine

**Keywords:** archaea, CRISPR-Cas, chronic viruses, coevolution, mutualism, symbiosis, transmission mode, virus-host interactions

## Abstract

Theory, simulation, and experimental evolution demonstrate that diversified CRISPR-Cas immunity to lytic viruses can lead to stochastic virus extinction due to a limited number of susceptible hosts available to each potential new protospacer escape mutation. Under such conditions, theory predicts that to evade extinction, viruses evolve toward decreased virulence and promote vertical transmission and persistence in infected hosts. To better understand the evolution of host-virus interactions in microbial populations with active CRISPR-Cas immunity, we studied the interaction between CRISPR-immune Sulfolobus islandicus cells and immune-deficient strains that are infected by the chronic virus SSV9. We demonstrate that Sulfolobus islandicus cells infected with SSV9, and with other related SSVs, kill uninfected, immune strains through an antagonistic mechanism that is a protein and is independent of infectious virus. Cells that are infected with SSV9 are protected from killing and persist in the population. We hypothesize that this infection acts as a form of mutualism between the host and the virus by removing competitors in the population and ensuring continued vertical transmission of the virus within populations with diversified CRISPR-Cas immunity.

## Abstract (precis)

Multiple studies, especially those focusing on the role of lytic viruses in key model systems, have shown the importance of viruses in shaping microbial populations. However, it has become increasingly clear that viruses with a long host-virus interaction, such as those with a chronic lifestyle, can be important drivers of evolution and have large impacts on host ecology. In this work, we describe one such interaction with the acidic crenarchaeon Sulfolobus islandicus and its chronic virus *Sulfolobus* spindle-shaped virus 9. Our work expands the view in which this symbiosis between host and virus evolved, describing a killing phenotype which we hypothesize has evolved in part due to the high prevalence and diversity of CRISPR-Cas immunity seen in natural populations. We explore the implications of this phenotype in population dynamics and host ecology, as well as the implications of mutualism between this virus-host pair.

## INTRODUCTION

Viruses can play influential roles in population dynamics, ecology, and evolution in all three domains of life. Most studies of viruses of microbes have focused on the negative effects of viral infection, in part due to the study of primarily lytic viruses which must kill their host in order to reproduce successfully. However, there has recently been a greater appreciation for the beneficial, mutualistic interactions between virus and host and the coevolutionary forces that may shift this symbiosis from antagonistic to mutualistic (1–4). Chronic viruses are transmitted vertically from mother to daughter cell. Unlike their lytic and temperate counterparts, chronic viruses can also be transmitted horizontally by producing new virions without killing their hosts (often through budding) (1, 4). Because of this mixed mode of transmission, selection can favor the vertical or horizontal mode depending on factors such as the availability of susceptible hosts and costs associated with viral carriage (1, 5, 6).

We have shown that highly active and diverse CRISPR-Cas immunity can create conditions of low-susceptibility host density in which lytic viruses can go extinct (7). These conditions are defined as distributed immunity, where CRISPR-immune hosts have a diversity of spacers targeting a virus, thus leading to a decreased probability of a single escape mutation by the virus leading to the emergence of a viral epidemic of the population (8, 9). Under these conditions, nonproductive infection of CRISPR-immune hosts leads to degradation of viral genomes and therefore comes at a significant cost to the virus’s fitness (7, 10–13). We predict that conditions of highly distributed immunity would result in a shift toward vertical transmission of chronic viruses. We have identified the structure of distributed immunity that is maintained over time in the natural geographically isolated population of the archaeon Sulfolobus islandicus from Kamchatka, Russia (14).

**FIG 1:**
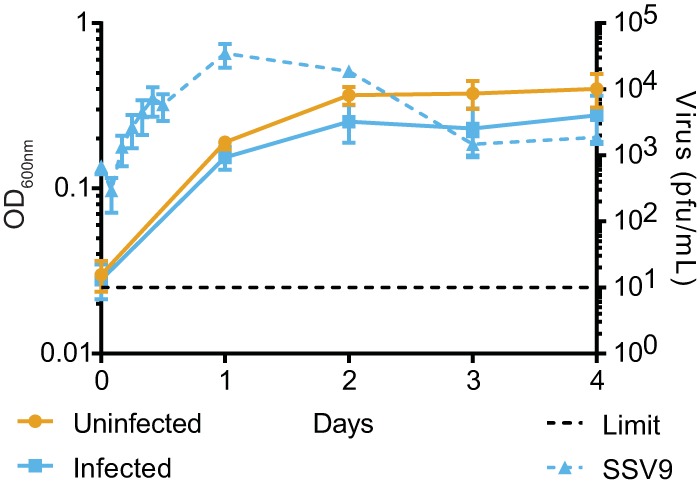
Challenge with SSV9 yields isolated chronically infected S. islandicus strains. Growth of an isolated, chronically infected strain (Δ*cas6*:SSV9.1, solid blue line) and an uninfected isogenic strain (Δ*cas6* strain, gold line), as monitored by optical density. Throughout the growth curves, SSV9 production (blue dashed line) was monitored in the chronically infected strain by plaque assay. Error bars show mean results ± standard errors of the means (SEM) (*n* = 3).

We used viruses isolated from both Kamchatka and Yellowstone National Park, USA, populations to investigate their interactions with immune hosts and whether they evolve mechanisms to persist in host populations with highly diversified CRISPR-Cas immunity (14–16). Through plaque assays with enrichment supernatants and a diversity of 11 wild S. islandicus hosts from Yellowstone and Kamchatka, the primary viruses that we isolated from the Kamchatka population are *Sulfolobus* spindle-shaped viruses (SSVs) from the *Fuselloviridae* family (17). Schleper et al. first discovered SSVs as chronic viruses that have a circular double-stranded DNA genome that can be maintained in a population through integration into the host genome or be kept as an episome, both of which are transmitted vertically (18). SSVs continuously bud from host cells without killing them at a rate of approximately 1 PFU for every 10,000 cells and can be transmitted horizontally to a new host (Fig. 1) (19). We have demonstrated experimentally that the CRISPR-Cas system can actively prevent productive SSV9 infection and that in this system, exposure of viral supernatants to the host causes dormancy (17). However, while this study suggests a possible infection-independent response of the challenged host, it lacks the context of population dynamics and a chronically infected host. Within the population context, it has been shown that Kamchatka population genomes show a high level of population-distributed immunity to SSVs (15). This results in very few susceptible hosts and a probability of evolutionary emergence of a successful viral escape variant on the scale of 10^−4^ (see Fig. S1 in the supplemental material) (8, 9, 14, 15). Here, we test the virus-host interaction and competitive host fitness when the virus competes against cells that are CRISPR immune and susceptible. In doing so, we uncover a novel antagonistic interaction between chronically infected strains and uninfected counterparts that we hypothesize drives a mutualism between viruses and their archaeal hosts in populations with distributed immunity.

10.1128/mBio.00404-20.1 FIG S1 Probability of emergence of SSV9 from a CRISPR-immune population. Evolutionary emergence of an escape SSV9 virus from a CRISPR-immune population based on equations found in the work of H. Chabas, S. Lion, A. Nicot, S. Meaden, et al. (PLoS Biol 16:e2006738, 2018, https://doi.org/10.1371/journal.pbio.2006738). Variables for SSV9 used were a birth rate of 0.1165 virus/CFU/h at exponential phase of growth for a chronically infected strain, a death rate of 0.6173 virus/h based on negative controls of an adsorption assay (M. A. Bautista, C. Zhang, and R. J. Whitaker, mBio 6:e02565-14, 2015, https://doi.org/10.1128/mBio.02565-14), a population immunity of 0.56, and 16 immune alleles based on the work by M. D. Pauly, M. A. Bautista, J. A. Black, and R. J. Whitaker (Philos Trans R Soc Lond B Biol Sci 374:20180093, 2019, https://doi.org/10.1098/rstb.2018.0093), and a mutation rate of 10^−6^. Download FIG S1, EPS file, 1.6 MB. Copyright © 2020 DeWerff et al. 2020 DeWerff et al. This content is distributed under the terms of the Creative Commons Attribution 4.0 International license.

## RESULTS AND DISCUSSION

### Viral challenge with SSV9 leads to chronic infection in immune-deficient strains.

The CRISPR-immune strain RJW003 is derived from type strain M.16.4 (isolated from Kamchatka, Russia) and has a perfect spacer match to that of SSV9 (also from Kamchatka), which is sufficient for CRISPR-Cas immunity and prevention of viral infection (see Table S1 in the supplemental material) (17). This strain is marked with a deletion at the *lacS* locus, which is neutral in this context but allows identification in competition with other strains through colony staining or quantitative PCR (qPCR) techniques (Fig. S3). We created a CRISPR immunity-deficient strain of our Sulfolobus islandicus Δ*cas6* type strain (RJW002 Δ*cas6*) and infected it with SSV9 (17). Chronically infected cells can grow while new viral particles are being shed but have a slight cost in final optical density compared to those of uninfected isogenic strains in stationary phase. The peak of viral production occurs during exponential phase (Fig. 1). The chronically infected strain was sequenced to determine integration of the SSV9 genome; however, read mapping analysis supported the finding that the virus was being maintained as a nonintegrated episome at a 6-fold copy number relative to the number on the host chromosome.

10.1128/mBio.00404-20.9 TABLE S1 Strains used in this study. Download Table S1, DOCX file, 0.02 MB. Copyright © 2020 DeWerff et al. 2020 DeWerff et al. This content is distributed under the terms of the Creative Commons Attribution 4.0 International license.

### Chronically infected strains outcompete immune strains.

**FIG 2:**
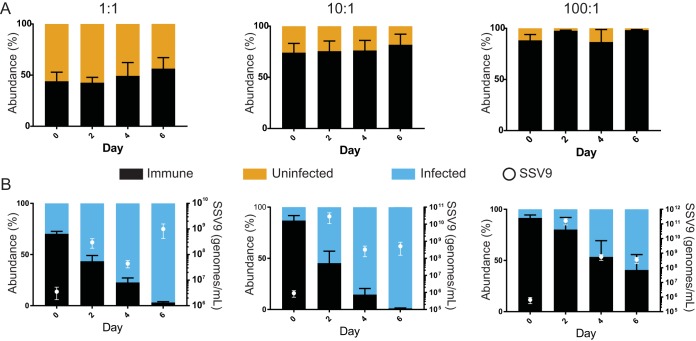
The chronically infected strain outcompetes the immune strain and can invade when chronically infected cells are rare. Relative abundances in competitions between a CRISPR-immune strain (RJW003, black) and an uninfected strain (Δ*cas6* strain, gold) (A) or between a CRISPR-immune strain and a chronically infected strain (Δ*cas6*:SSV9.1, blue) (B) at initial frequencies of 1:1, 10:1, and 100:1. Strains grown to mid-log phase were mixed and monitored for 6 days by qPCR for host cell type. Days 2 and 4 show relative abundances before a 1:5 dilution into fresh medium. Error bars show mean results ± SEM (*n* = 3). Presence of virus (SSV9, white circles) in competitions of 1:1, 10:1, and 100:1 dilutions of chronically infected strains as monitored by qPCR of whole culture aliquots. Error bars show mean results ± SEM (*n* = 3).

To quantify the cost of viral infection, we tested the relative fitnesses of the chronically infected and uninfected isogenic strains in competition with RJW003, the CRISPR-Cas-immune Δ*lacS* genetic derivative. We predicted that the immune strain would outcompete the chronically infected strain due to its ability to prevent viral infection and the cost of infection. We found that the immune strain had a relative fitness above that of an uninfected CRISPR-Cas knockout strain in competition, suggesting that CRISPR-Cas is beneficial and not costly in this genetic background (Fig. S2). In contrast to our prediction, the chronically infected strain outcompeted the immune strain (Fig. 2) and had a higher selection rate coefficient for immune cells than the immune-deficient, uninfected isogenic control strain (0.594 ± 0.064/day and −0.374 ± 0.154/day for infected and uninfected strains, respectively; *P* value = 0.001). This fitness advantage is independent of which strain carries the *lacS* deletion (Fig. S4). Additionally, this competitive benefit still occurs when chronically infected cells are initially as rare as 1:100 (Fig. 2).

10.1128/mBio.00404-20.2 FIG S2 An immune strain outcompetes CRISPR immunity-deficient strains. (A) Growth curve of a CRISPR-immune strain (RJW003, black) and a CRISPR deletion strain (ΔCRISPRs, green) over 5 days as monitored by optical density. Error bars show mean results ± SEM (*n* = 3). (B) Relative abundances in a representative competition of a CRISPR-immune strain (RJW003, black) and a CRISPR-*cas* deletion strain (ΔCRISPRs, green) over 4 days without transfers and monitored by qPCR. Error bars show mean results ± SEM of one technical triplicate. Download FIG S2, EPS file, 1.5 MB. Copyright © 2020 DeWerff et al. 2020 DeWerff et al. This content is distributed under the terms of the Creative Commons Attribution 4.0 International license.

10.1128/mBio.00404-20.3 FIG S3 *lacS* locus to determine relative host abundance. (A) Representative image of cell growth and X-Gal staining efficiency of strains without the *lacS* locus (RJW003) and with the *lacS* locus (Δ*cas6* strain and Δ*cas6*:SSV9.1). (B) Specificity of qPCR targeted to the *lacS* locus or the junction of the deletion in the Δ*lacS* strains. Genomic DNA extracted from a Δ*lacS* strain (RJW003) or a *lacS*-positive strain (Δ*cas6* strain) were used. (C) Representative results of relative abundances in a competition between an immune strain (RJW003, black) and a uninfected strain (Δ*cas6*, gold) or a chronically infected strain (Δ*cas6*:SSV9.1, blue). Strains grown to mid-log phase were mixed and monitored for 4 days through X-Gal staining of CFUs for host cell type. Days 2 and 4 show relative abundances before a 1:5 dilution into fresh media. (D) Representative results of serial dilutions of competitions between an immune strain (RJW003, white colonies) and an uninfected (Δ*cas6* strain) or a chronically infected (Δ*cas6*:SSV9.1) strain, both of which stain blue. Download FIG S3, EPS file, 1.7 MB. Copyright © 2020 DeWerff et al. 2020 DeWerff et al. This content is distributed under the terms of the Creative Commons Attribution 4.0 International license.

10.1128/mBio.00404-20.4 FIG S4 A competitive advantage is independent of the *lacS* marker. Relative abundances in competitions between an immune strain (RJW002, grey) and an uninfected strain (RJW003 Δ*cas6*, gold) or a chronically infected strain (RJW003 Δ*cas6*:SSV9.3, blue). Strains grown to mid-log phase were mixed and monitored for 6 days by qPCR for host cell type. Days 2 and 4 show relative abundances before a 1:5 dilution into fresh medium. Error bars show mean results ± SEM (*n* = 3). Download FIG S4, EPS file, 1.5 MB. Copyright © 2020 DeWerff et al. 2020 DeWerff et al. This content is distributed under the terms of the Creative Commons Attribution 4.0 International license.

**FIG 3:**
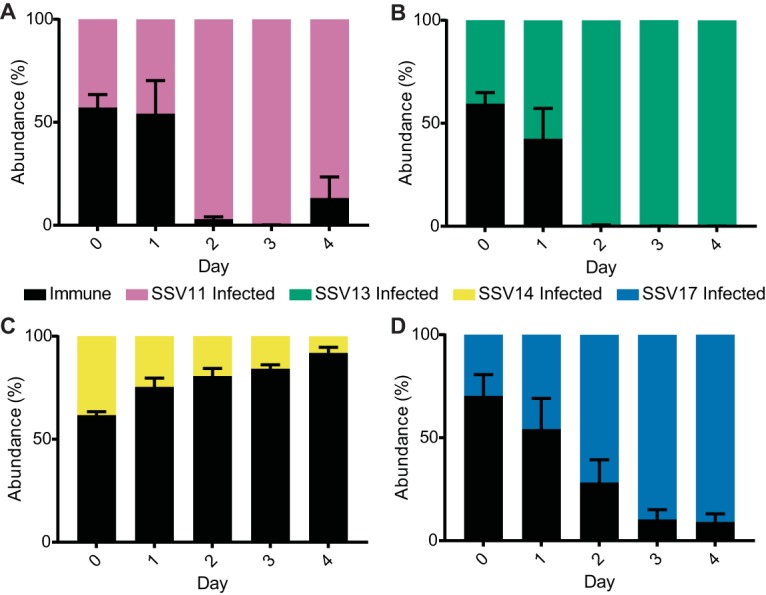
Competitive advantage is not specific to SSV9. Relative abundances in competitions between the immune strain (RJW003, black) and immune-deficient (Δ*cas6* background) strains infected with related SSVs: SSV11 (A), SSV13 (B), SSV14 (C), and SSV17 (D). Chronically infected strains were added to mid-log phase of an immune culture and monitored by qPCR for 4 days without dilutions with fresh medium. Error bars show mean results ± SEM (*n* = 3).

SSVs are a common type of virus found among the Kamchatka population; therefore, we isolated new strains chronically infected with related viruses to determine if this competitive advantage is specific to SSV9 or a broad phenotype. We isolated chronically infected strains that were infected with other SSVs isolated from both the Kamchatka region (SSV13, SSV14, and SSV17) and Yellowstone National Park (SSV11) (Table S1). Competition between the immune strain and these chronically infected strains shows that this competitive benefit is not specific to SSV9-infected cells but can be seen with viruses from both locations (Fig. 3). Shared open reading frames between these SSVs and SSV9 were compared with tblastn, revealing 1 gene sharing the same presence-absence pattern of the killing phenotype; however, competition with a chronically infected strain containing a deletion of this gene still showed a killing phenotype (Fig. S5) (20).

**FIG 4:**
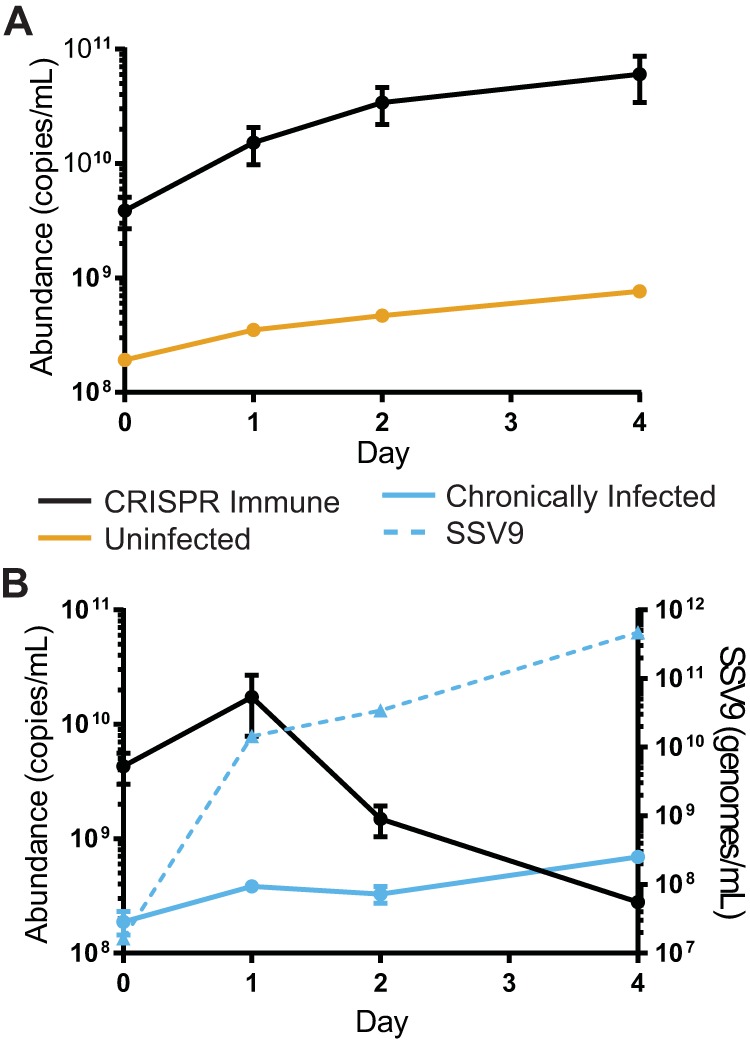
A chronically infected strain is antagonistic to an immune strain. Relative abundances in competitions between a CRISPR-immune strain (RJW003, black) and an uninfected strain (Δ*cas6* strain, gold) (A) or between a CRISPR-immune strain and a chronically infected strain (Δ*cas6*:SSV9.1, blue) (B). Uninfected or chronically infected strains were added to the mid-log phase of an immune culture and monitored by qPCR for 4 days without dilutions with fresh medium. In a representative experiment of three biological replicates, error bars show mean results ± SEM (3 technical replicates). Virus abundance was measured by qPCR (dashed line).

10.1128/mBio.00404-20.5 FIG S5 A genomic comparison of SSVs does not provide insight into a possible killing factor. (A) Presence and absence of SSV9 genes in other related SSVs: SSV11, SSV13, SSV14, and SSV17. The E81 gene (red box) matches the pattern of phenotypes seen in Fig. 4, suggesting that it may be the killing factor. (B) To test whether E81 has a role in the killing phenotype, a competition was completed with the chronically infected strain RJW002:SSV9.2 (dark blue), which carries a 7-kb genome deletion that includes the E81 gene. This deletion includes the protospacer, which has a 100% spacer match to the CRISPR array in M.16.4 and its derivatives. To test for killing activity without infection, this strain was competed with a virus-resistant strain, a ΔPibD strain (gray). Results show that this strain can be outcompeted by a chronically infected strain, and this is not dependent on the presence of the E81 gene in the virus. Strains grown to mid-log phase were mixed and monitored for 6 days by qPCR for host cell type. Days 2 and 4 show relative abundances before a 1:5 dilution into fresh medium. Error bars show mean results ± SEM (*n* = 2). Download FIG S5, EPS file, 2.2 MB. Copyright © 2020 DeWerff et al. 2020 DeWerff et al. This content is distributed under the terms of the Creative Commons Attribution 4.0 International license.

To test whether fitness advantage resulted from the faster growth of infected strains, we quantified the CFU of competing strains. As shown in Fig. 4, there was a decrease in the immune population after 24 h of coincubation at a 10:1 initial ratio, despite the fact that the population was initially in exponential growth, with an average doubling time of 14.2 h, suggesting that immune cells die and do not simply grow more slowly than infected cells. When looking at serial dilutions of the competing colonies, there was a mix of immune and chronically infected colonies seen on the initial day, but by day 1, there was evidence of only the infected strain, although it had not yet reached carrying capacity (Fig. S3). This killing effect would also explain the anomalous plaque formation observed with chronic viruses that do not kill their hosts (18). We hypothesize that the direct killing of uninfectable cells is a benefit for the virus, as it removes potentially nonpermissive CRISPR immune hosts and prevents particle loss to CRISPR-Cas targeting, while the chronically infected host remains unaffected. This phenotype may explain some of the cost of infection (Fig. 1), as segregants are killed when they lose the virus. In this emergent mutualism, infection also benefits the host cell in competition with immune cells. This is an advantage in a metapopulation like that found in Sulfolobus islandicus strains from Yellowstone National Park (21), in which invasion of local environments that have immunity can increase host fitness.

### Virus-mediated antagonism is mediated by a proteinaceous killing factor.

**FIG 5:**
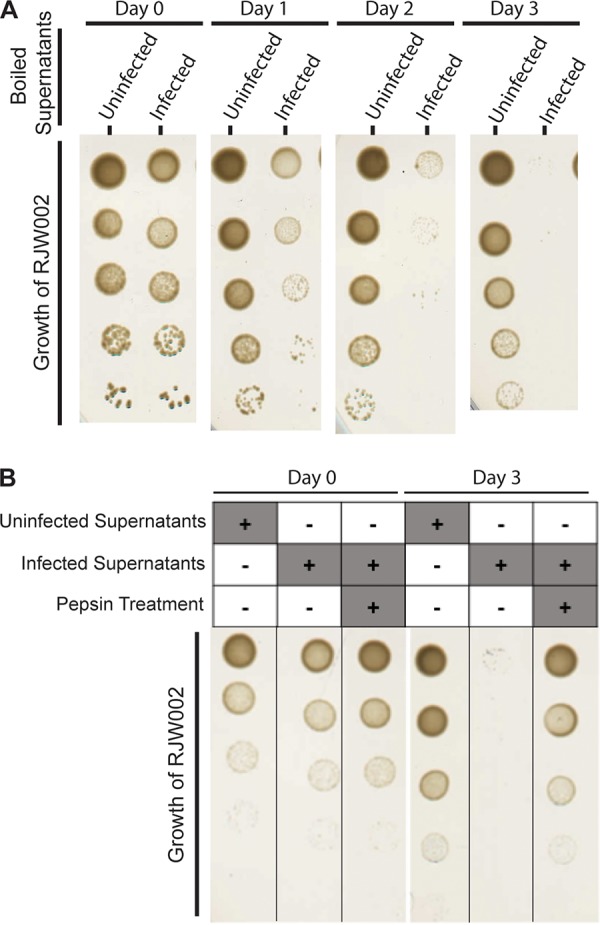
Infection-mediated antagonism does not require active infection. (A) Representative images of CRISPR-immune strain RJW002 growth after incubation with boiled cell-free supernatants from an uninfected (Δ*cas6* strain) or a chronically infected (Δ*cas6*:SSV9.1) strain. Immune cells were dosed with PFU-free boiled supernatants on days 0, 1, and 2. (B) Representative images of CRISPR-immune strain RJW002 growth on days 0 and 3 after incubation with boiled cell-free supernatants from an uninfected (Δ*cas6* strain) or a chronically infected (Δ*cas6*:SSV9.1) strain with and without pepsin treatment. Immune cells were dosed with PFU-free boiled treated or untreated supernatants on days 0, 1, and 2.

To determine the mechanism of antagonism, we tested whether killing is due to active infection of immune cells by SSV9. Cell-free supernatants were collected by filtration and boiled for 30 min; after this time, we determined the titers of untreated and boiled samples, and boiled samples were shown to have infectious units below the limit of detection (Fig. S4). As shown in Fig. 5, at 24 h, there was a severe loss of viability of the cells exposed to PFU-free supernatants from the chronically infected strain, in contrast to that of controls, and this viability remained low throughout the experiment. This demonstrates that infectious particles and active infection are not needed for the infected-cell antagonism on uninfected cells, making this phenotype clearly distinct from some dormancy phenotypes which may produce the similar result of immune cell death by activation of CRISPR targeting of host transcripts seen with Cas13 targeting in Listeria ivanovii (22). However, this may explain previous results in the Sulfolobus islandicus and SSV9 system which suggested that the presence of virus, even if inactivated by UV, can cause a stall in host growth. Here, we show that this phenotype in the context of the mixed population is the basis of a benefit for both the virus and the chronically infected strain. This extracellular killing mechanism is also different from those of abortive infection systems that can also utilize toxin-antitoxin systems due to the killing activity not requiring active infection (23). Our data show that killing is not contact mediated, that it can occur without the presence of infected cells, and that a killing factor would be extremely stable in an acidic hot spring environment compared to in a viral particle.

To test the nature of the killing factor, we treated cell-free supernatants from an uninfected and a chronically infected strain with the protease pepsin overnight. After 3 doses, it was found that the immune strain was still viable if challenged with the pepsin-treated supernatants or the supernatants from the uninfected strain (Fig. 5B). From this, we suggest that the mechanism of action is a toxin secreted into the medium similar in mechanism to that of killer yeast strains, where infection with an RNA virus causes the production and secretion of a protein toxin (24). Addictive plasmids evolved a similar method of utilizing a toxin for ensuring vertical transmission; however, it is not exported from the cell (25, 26). The identity of this toxin or whether it is virus or host encoded is unknown at this time. The proteinaceous toxins (sulfolobicins) specific to this genus that have been isolated from vesicles from *Sulfolobus* species were not encoded by the virus or the M.16.4 background (27, 28). Chronically infected supernatants concentrated to a 4× relative concentration by tangential-flow filtration through a 30,000 molecular-weight-cutoff (MWCO) filter and boiled still show killing activity and growth inhibition (Fig. S8). The activity of the concentrated supernatants suggests that the killing factor is larger than a 30,000 MWCO, as it is not lost in the filtration process.

### The killing phenotype selects for successful horizontal transmission.

**FIG 6:**
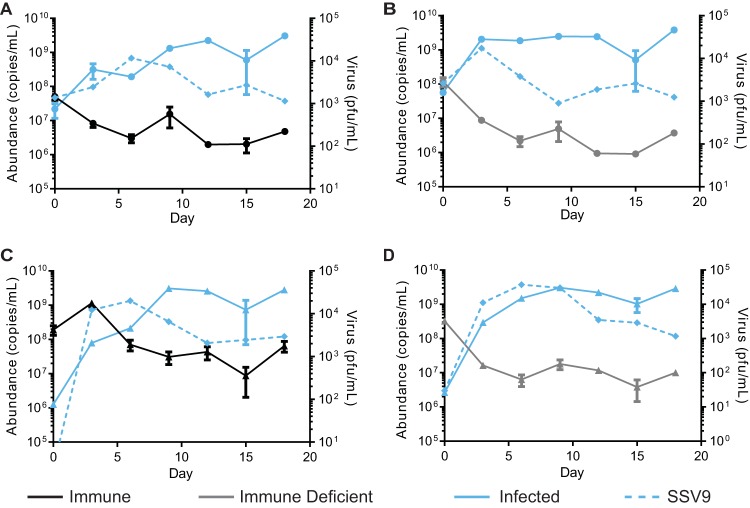
Persistence of immune and immune-deficient strains in competition. Relative abundances in competitions between the CRISPR-immune strain (A and C) or an immune-deficient strain (B and D), with the chronically infected strain at a ratio of 1:1 (A and B, circles) or 1:100 (C and D, triangles). Strains were grown to mid-log phase and mixed before being sampled for host abundance by qPCR. Supernatants were saved, and PFU were quantified by plaque assay to determine the amount of the virus (right axis). Every 3 days, the culture was sampled and then diluted 1:5 in fresh medium. Shown are the results of a representative experiment of three biological replicates; error bars show mean results ± SEM (3 technical replicates). Virus abundance was measured by qPCR (dashed line).

Since chronically infected cells continue to grow while producing the extracellular killing factor, we hypothesize that the virus encodes a rescue factor or antitoxin that makes chronically infected cells resistant to its effects. This would provide a screening mechanism to promote new horizontal infections established in nonimmune cells while limiting the loss of particles to CRISPR-immune cells. To test this, we competed the chronically infected and uninfected strains with the CRISPR-Cas-immune strain or immune-deficient strain. If the killer phenotype selects for successful horizontal transmission, we predicted that the CRISPR-immune strain would not persist in a long-term competition with the chronically infected strain and that the immune-deficient strains would persist, as they could chronically infect cells through horizontal transmission (Fig. S6). However, we found instead that both the immune and the immune-deficient strains can persist throughout the competition and at similar levels, contrary to our hypothesis (Fig. 6). Samples throughout the competition were plated for colonies and stained with X-Gal (5-bromo-4-chloro-3-indolyl-β-d-galactopyranoside) to determine the genetic background. While obtaining well-isolated colonies of the tested population (initially immune or immune deficient) was rare, all colonies tested positive for the presence of SSV9 through colony PCR, indicating successful horizontal transmission.

10.1128/mBio.00404-20.6 FIG S6 Antagonism does not require active infection. (A) Cell-free supernatants were tested for infective particles by quantifying PFU before and after a 30-min boil. Results show that after a 30-min boil, the numbers of PFU fall below the limit of detection for the assay. Download FIG S6, TIF file, 4.9 MB. Copyright © 2020 DeWerff et al. 2020 DeWerff et al. This content is distributed under the terms of the Creative Commons Attribution 4.0 International license.

10.1128/mBio.00404-20.7 FIG S7 Model of killing and rescue factors selecting for successful horizontal transmission of the virus. Chronically infected cells produce both viral particles and stable killing factors. Cells that can establish chronic infection will produce a rescue factor that allows for the survival of the killing phenotype. CRISPR-*cas*-immune cells that prevent infection are selected against due to the action of the killing phenotype. Download FIG S7, EPS file, 0.8 MB. Copyright © 2020 DeWerff et al. 2020 DeWerff et al. This content is distributed under the terms of the Creative Commons Attribution 4.0 International license.

10.1128/mBio.00404-20.8 FIG S8 Killing activity is not specific to one host strain. Supernatants were collected by filtration and then concentrated by a 30,000-MWCO tangential filtration system to a relative concentration of 4×, and aliquots were boiled for 30 min. Concentrated supernatants were then spotted on lawns of related *Sulfolobaceae* strains: S. islandicus (RJW002, the Δ*cas6* strain, and M.16 strains), Sulfolobus acidocaldarius, and Sulfolobus solfataricus. Supernatants were spotted at 0, 18, and 24 h and incubated for a total of 3 days before being visualized. Representative images from three biological replicates are shown. Download FIG S8, EPS file, 1.9 MB. Copyright © 2020 DeWerff et al. 2020 DeWerff et al. This content is distributed under the terms of the Creative Commons Attribution 4.0 International license.

While this was expected for the immune-deficient population, this was surprising for the immune population. We hypothesize that this may be due to a CRISPR-Cas failure rate, evolution of viral escape mutants, or some combination of the two. Though surprising, this result supports the supposition that, while it seems that there is a strong vertical transmission component in this system, the possibility of a horizontal component should not be excluded, even for immune populations.

### A virus-resistant strain is outcompeted by a chronically infected strain.

**FIG 7:**
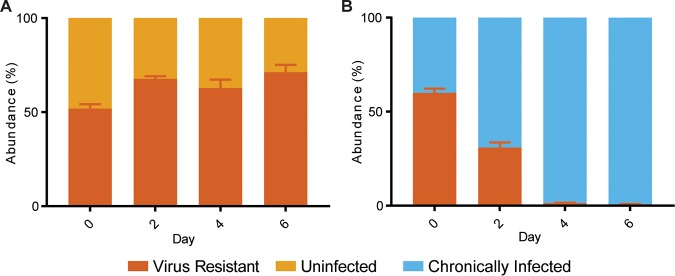
A viral-infection-resistant strain is outcompeted in a competition with a chronically infected strain. Shown are relative abundances in competitions between a virus-resistant strain (Δ6068) and an uninfected strain (RJW003 Δ*cas6*) (A) and between Δ6068 and a chronically infected strain (RJW003 Δ*cas6*:SSV9.3). Strains grown to mid-log phase were mixed and monitored for 6 days by qPCR for host cell type. Days 2 and 4 show relative abundances before a 1:5 dilution into fresh medium. Error bars show mean results ± SEM (*n* = 3).

To further test our hypothesis that active infection is not needed for killing but needed for survival, a virus-resistant strain was competed with a chronically infected strain. This virus-resistant strain was isolated from a viral challenge and found to have deletions of two surface pilin proteins needed to establish infection (20). When this strain is competed with a chronically infected strain, it is driven to almost extinction by day 6 (Fig. 7). Without the ability to establish chronic infection by horizontal transmission, the chronically infected strain has a larger competitive advantage over the resistant strain than the CRISPR-immune strain, with selection rates of 0.883 ± 0.015/day against the resistant strain and 0.594 ± 0.064/day against the CRISPR-immune strain, as stated earlier. Additionally, because the resistant strain is unable to be infected, this supports our earlier finding that active infection is not required for killing but instead for protection against toxin produced by other infected stains.

### Chronically infected supernatants show killing activity on contemporary Sulfolobus islandicus strains.

**FIG 8:**
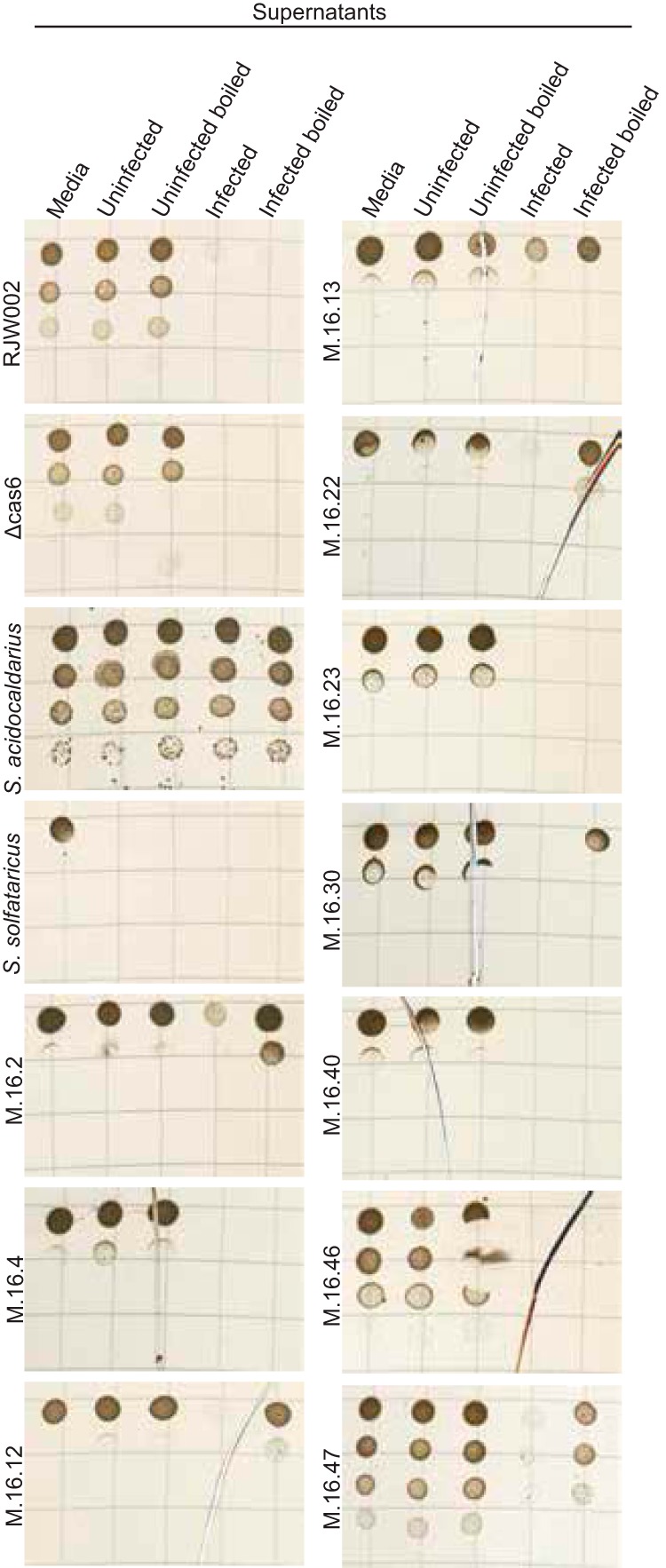
Treatment of chronically infected supernatants on related *Sulfolobaceae* strains shows decreased viability. S. islandicus (RJW002, Δ*cas6*, and M.16 strains), Sulfolobus acidocaldarius, and Sulfolobus solfataricus were grown in liquid media dosed with uninfected or chronically infected supernatants with and without prior boiling. Strains were incubated for 3 days and then serially diluted and spotted onto a solid medium to determine viability. Representative images from two biological replicates are shown.

Work until this point has been performed with a variety of strains within the same M.16.4 genetic background. However, if this is an emergent mutualism, it should not affect just one strain in a population that is known to be diverse. Therefore, killing activity was assessed on S. islandicus strains isolated from the same hot spring at the same time, as well as on two distantly related organisms, Sulfolobus acidocaldarius and Sulfolobus solfataricus P2 (29–33). Supernatants were collected from uninfected and chronically infected strains by filtration, and aliquots we boiled for 30 min to remove infectious particles. *Sulfolobus* strains were then dosed with media, uninfected supernatants, or chronically infected supernatants for 3 days and then spotted for viability. While one distantly related strain showed complete resistance to the virus and killing activity (S. acidocaldarius), there was a mix of phenotypes seen, suggesting that some strains may be susceptible to the killing activity and not to viral infection or, conversely, to viral infection but not the killing activity (Fig. 8). Additionally, strains were grown on lawns and spotted with 3 doses of relative 4×-concentrated samples as described earlier at 0, 18, and 24 h (Fig. S8). Supporting the cell viability spots, almost all strains show some growth inhibition from the chronically infected supernatants; however, this is less pronounced in some of the boiled supernatants, which supports the idea that the inhibition is due to virus infection and not killing by a toxin. Overall though, the killing activity of contemporary strains suggests that this phenotype may be a driving force in the ecology of acidic hot springs and that it promotes a strong mutualism between SSV9 and its hosts.

### Concluding remarks.

The population structure of susceptible hosts can drive the evolution of their viruses. While in some cases, this is theoretically predicted to select for decreases in virulence and increased vertical transmission, in Sulfolobus islandicus and SSV9, this interaction in the context of highly distributed immunity may select for a unique way of ensuring viral persistence by removing competing nonpermissive hosts. We have shown that strains with CRISPR-Cas immunity and viral resistance are subjected to this killing phenotype, suggesting that while active infection is not needed for killing, it may be needed to prevent killing. Our hypothesis is that the chronically infected cells produce a toxin that can be secreted and produce an antitoxin that remains within the infected cell. This would allow for specific targeting of the nonpermissive host that either degrades the viral genome through CRISPR-Cas immunity or prevents infection, as with potentially virus-resistant strains. This phenotype seems especially beneficial for a chronic virus with a mixed mode of transmission, as the continued production of viral particles would allow an uninfected, permissive host to be selected in the population, as well as remove competing strains to ensure vertical transmission. This is especially important in populations with high levels of distributed CRISPR-Cas immunity, where new viruses would most often be degraded, leading to viral extinction.

Together with the benefits to the infected host as shown in this study, we believe that this interaction may shift toward a mutualistic interaction between the virus and its host, and future studies into the mechanism and ecology of this interaction may further support the changing evolution of this symbiosis.

## MATERIALS AND METHODS

### Microbial strains.

All Sulfolobus islandicus strains (see Table S1 in the supplemental material) were grown in dextrin-tryptone medium supplemented with uracil (DTU), as needed (29, 34). Cultures were grown at 75°C without shaking. Viral stocks and supernatants were collected at exponential growth of a chronically infected strain, filtered through a 0.2-μm polyethersulfone (PES) bottle-top filter (Nalgene catalog number 595-4520), and then stored at 4°C.

### Infections with SSV viruses.

An S. islandicus immune-deficient ΔCas6 deletion strain was challenged with viruses as described previously (17). Following the 5-h incubation with virus filtrate, cells were resuspended in 70 ml DTU in a 75-cm^2^ culture flask and incubated at 75°C. Host growth was monitored, and after 8 days or when an optical density at 600 nm (OD_600_) of 0.10 was reached, cultures were plated in DTU-Gelrite overlays for colony isolation. To isolate a chronically infected strain after 10 to 14 days of incubation at 75°C, colonies were picked and resuspended in DTU and tested for infection using 16S rRNA- and SSV-specific primers. Colonies that tested positive for infection were then grown, supernatants were collected, and 10 μl was spotted on a lawn of the strain S. islandicus Y08.82.36 for production of PFU.

### Library prep and sequencing.

Genomic libraries were prepared for all viruses using the Nextera XT kit (Illumina) according to the manufacturer's instructions. Libraries were pooled and sequenced using paired-end MiSeq version 2.5 by the W. M. Keck Center for Comparative and Functional Genomics at the University of Illinois at Urbana-Champaign. Reads were quality filtered using FASTX-Toolkit, and adapters were trimmed using Cutadapt (35). Host genome assemblies were compared to those of host strain M.16.4 using breseq (36).

### Determining SSV integration status.

Quality filtered reads from a chronically infected strain were mapped to the SSV9 integrase by using bwa (37). Read names were extracted, and the mate pairs of mapped reads were then pulled into a separate file. Read mates were mapped back to the S. islandicus M.16.4 genome using bwa, and the alignment was visualized using Geneious. Areas of read coverage of the mate pairs were analyzed, and we found no evidence of reads mapping to the host genome above our map quality score threshold.

### Growth curves.

To compare levels of growth of the uninfected and infected strains, cells were collected by centrifugation at 4,000 × *g* for 20 min. Cells were resuspended in fresh DTU and then diluted to a final volume of 20 ml at an initial OD_600_ of 0.04 and incubated without shaking at 75C. Cell growth was determined by measuring optical density every 24 h. At the time of sampling, 500-μl aliquots were taken and culture supernatants were collected by centrifugation at 15,000 × *g* in a microcentrifuge. Collected supernatants were then used for a plaque-forming assay. One hundred microliters of the supernatant was mixed with 500 μl of a 10×-concentrated strain, S. islandicus Y08.82.36. This mixture was incubated at 75°C for 30 min, and then 5 ml of an overlay sucrose-yeast (SY) medium was poured on SY plates as previously described (18). Plates were incubated at 75°C for 48 h.

### Transfer competitions.

Strains used in competitions were grown to mid-log phase (OD_600_ of 0.10 to 0.18). Cells were collected by centrifugation at 4,000 × *g* for 20 min and resuspended in fresh media. Cells were then inoculated at the respective ratio of each type so that there was approximately 3 × 10^9^ combined total cells. Cultures were incubated for 2 days at 75°C without shaking; after 2 days, the culture was diluted 1:5 into fresh medium. This was repeated 2 more times, for a total competition duration of 6 days. At the start of the competition and before and after dilution, 200 μl of culture was collected and frozen for analysis of cell abundance by qPCR and 200 μl of supernatant was collected for analysis of viral abundance (see Table S3 for primers). Primers targeting a 150-bp portion within the *lacS* gene or the residues left after *lacS* deletion were used to distinguish cell types using a previously described qPCR protocol. Standard curves were created using extracted genomic DNA from purified strains with and without the *lacS* gene. In the long-term transfer competitions, the experiment was conducted in a similar manner, but cells were transferred every 3 days.

### Nontransfer competitions.

Strains used in competitions were grown to mid-log phase (OD_600_, 0.10 to 0.18). Cells were collected by centrifugation at 4,000 × *g* for 20 min and resuspended in fresh media. Cells were then inoculated at the respective ratio of each type so that there were approximately 3 × 10^9^ total combined cells. Cultures were incubated at 75°C for 4 days, and 200 μl of culture was collected every day and frozen for analysis of cell abundance by qPCR as described above.

### Heat-killed supernatant assays.

Cultures of the chronically infected strain or the isogenic uninfected strain were grown to an optical density of 0.15 to 0.20 and then filtered through a 0.22-μm PES filter. Supernatants were then placed in boiling water for 30 min. Supernatant samples from before and after boiling were tested for infectious particles by plaque assay as described above. When pepsin was used, the treatment of the supernatants was completed with immobilized pepsin (Thermo Scientific catalog number 20343) according to the manufacturer’s instructions and incubated at 37°C overnight in a rotator. Strains tested were grown to mid-log phase and then diluted to an OD_600_ of 0.03 in fresh medium, the boiled supernatants, or the unboiled supernatants. At 24, 48, and 72 h, cultures were centrifuged, and cells were resuspended in fresh doses of medium or the supernatants. Every 24 h, cell viability was tested by spotting 10-μl cell dilutions onto DTU plates.

10.1128/mBio.00404-20.10 TABLE S2 Primers used in this study. Download Table S2, DOCX file, 0.01 MB. Copyright © 2020 DeWerff et al. 2020 DeWerff et al. This content is distributed under the terms of the Creative Commons Attribution 4.0 International license.
