# Supplementary material for: Killer Archaea: Virus-Mediated Antagonism to CRISPR-Immune Populations Results in Emergent Virus-Host Mutualism
Source: mBio. 2020 Apr 28;11(2):e00404-20. doi: 10.1128/mBio.00404-20 (PMC7188992; doi:10.1128/mBio.00404-20)
Supplement: TABLE S1 [file mBio.00404-20-st001.docx]

**Table S1. Strains used in this study**

| **Strain** | **Genotype/Description** | **Phenotype/Description** | **Reference** |
| --- | --- | --- | --- |
| RJW002 | M.16.4Δ*pyrEF* | CRISPR immune to SSV9. Uracil auxotroph. | [34] |
| RJW003 | M.16.4Δ*pyrEF*Δ*lacS* | Derivative of RJW002 without B-galactosidase | [34] |
| Δcas6 | M.16.4Δ*pyrEF*Δ*cas6* | Immune deficient – crRNA processing inactivated; uninfected; uracil auxotroph | [17] |
| RJW003Δcas6 | M.16.4Δ*pyrEF*Δ*lacS*Δ*cas6* | Derivative of Δcas6 without B-galactosidase | This work |
| Δcas6:SSV9.1 | M.16.4Δ*pyrEF*Δ*cas6* + SSV9 | Chronically infected with full length SSV9; Derivative of Δcas6 | This work |
| RJW002:SSV9.2 | M.16.4Δ*pyrEF* + SSV9.2Δ7kb | Chronically infected with SSV9 containing a 7kb deletion; Derivative of RJW002 | [20] |
| RJW003Δcas6:SSV9.3 | M.16.4Δ*pyrEF*Δ*lacS*Δ*cas6* + SSV9 | Chronically infected with full length SSV9; Derivative of RJW003Δcas6 | This work |
| Δcas6:SSV11 | M.16.4Δ*pyrEF*Δ*cas6* + SSV11 | Chronically infected with SSV11; Derivative of Δcas6 | This work |
| Δcas6:SSV13 | M.16.4Δ*pyrEF*Δ*cas6* + SSV13 | Chronically infected with SSV13; Derivative of Δcas6 | This work |
| Δcas6:SSV14 | M.16.4Δ*pyrEF*Δ*cas6* + SSV14 | Chronically infected with SSV14; Derivative of Δcas6 | This work |
| Δcas6:SSV17 | M.16.4Δ*pyrEF*Δ*cas6* + SSV17 | Chronically infected with SSV17; Derivative of Δcas6 | This work |
| Δ6068 | M.16.4Δ*pyrEF*Δ*A1*Δ*6068* | Resistant to SSV9 – deletion of 6kb region including 2 pilins; Immune deficient – deletion of A1 CRISPR array; uracil auxotroph | [20] |
| ΔpibD | M.16.4Δ*pyrEF*Δl*acS*Δ*argD*, *pibD::argD* | Resistant to SSV9 – deletion of pre-pilin processing gene; | [20] |
| M.16.2 | Strain isolated from M16 hot spring, Mutnovsky region, Kamchatka, RUS | Immune to SSV9 | [29,30] |
| M.16.4 | Strain isolated from M16 hot spring, Mutnovsky region, Kamchatka, RUS | Immune to SSV9 | [29,30] |
| M.16.12 | Strain isolated from M16 hot spring, Mutnovsky region, Kamchatka, RUS | Resistant to SSV9 | [29,30] |
| M.16.13 | Strain isolated from M16 hot spring, Mutnovsky region, Kamchatka, RUS | Immune to SSV9 | [29,30] |
| M.16.22 | Strain isolated from M16 hot spring, Mutnovsky region, Kamchatka, RUS | Resistant to SSV9 | [29,30] |
| M.16.23 | Strain isolated from M16 hot spring, Mutnovsky region, Kamchatka, RUS | Susceptible to SSV9 | [29,30] |
| M.16.27 | Strain isolated from M16 hot spring, Mutnovsky region, Kamchatka, RUS | Immune to SSV9 | [29,30] |
| M.16.30 | Strain isolated from M16 hot spring, Mutnovsky region, Kamchatka, RUS | Susceptible to SSV9 | [29,30] |
| M.16.40 | Strain isolated from M16 hot spring, Mutnovsky region, Kamchatka, RUS | Susceptible to SSV9 | [29,30] |
| M.16.43 | Strain isolated from M16 hot spring, Mutnovsky region, Kamchatka, RUS | Immune to SSV9 | [29,30] |
| M.16.46 | Strain isolated from M16 hot spring, Mutnovsky region, Kamchatka, RUS | Immune to SSV9 | [29,30] |
| M.16.47 | Strain isolated from M16 hot spring, Mutnovsky region, Kamchatka, RUS | Susceptible to SSV9 | [29,30] |
| *Sulfolobus acidocaldarius* | DSM 639 |  | [31] |
| *Silfolobus solfataricus* P2 | DSM 1617 |  | [32,33] |
