# Supplementary figures and images for: Killer Archaea: Virus-Mediated Antagonism to CRISPR-Immune Populations Results in Emergent Virus-Host Mutualism
Source: mBio. 2020 Apr 28;11(2):e00404-20. doi: 10.1128/mBio.00404-20 (PMC7188992; doi:10.1128/mBio.00404-20)

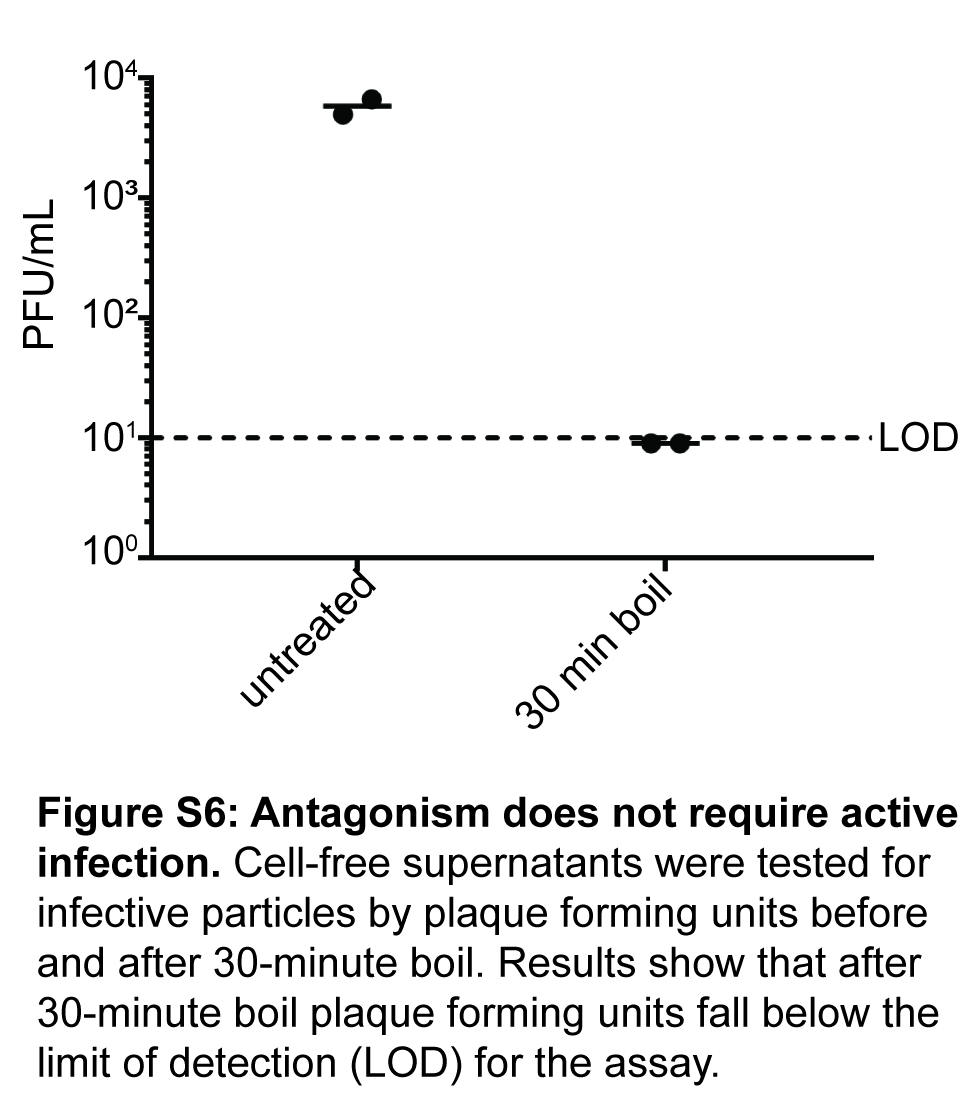

Supplement: FIG S6 [file mBio.00404-20-sf006.tif]
