# Supplementary material for: Killer Archaea: Virus-Mediated Antagonism to CRISPR-Immune Populations Results in Emergent Virus-Host Mutualism
Source: mBio. 2020 Apr 28;11(2):e00404-20. doi: 10.1128/mBio.00404-20 (PMC7188992; doi:10.1128/mBio.00404-20)
Supplement: TABLE S2 [file mBio.00404-20-st002.docx]

**Table S2. Primers used in this study**

| **Name** | **Sequence (5’ to 3’)** | **Reference** |
| --- | --- | --- |
| SSV9 qPCR Forward | GTGAAGCGACCAACATAGGTGCAA | [17] |
| SSV9 qPCR Reverse | GTTGCGTTTGTACCGGTTACGCTA | [17] |
| ΔlacS qpcr Forward | GACTTCCCAATTAGATTAACGTTAAGTGGTG | This work |
| ΔlacS qpcr Reverse | CTTTAGGTTCGGTTGGTCTCTGCAG | This work |
| LacS positive qPCR Forward | GCCTGGTCCGTTTTCTGGG | This work |
| LacS positive qPCR Reverse | CGGTTGGTCTCAAGCCGG | This work |
